# Promising Novel Method of Acetylation Modification for Regulating Fatty Acid Metabolism in *Brassica napus* L.

**DOI:** 10.3390/biology11040483

**Published:** 2022-03-22

**Authors:** Xiaojiang Jia, Xinghua Xiong, Hao Chen, Gang Xiao, Qian Cheng, Zhenqian Zhang

**Affiliations:** 1 College of Agriculture, Hunan Agricultural University, Changsha 410128, China; jxjlxy@126.com (X.J.); xiongene@hunau.edu.cn (X.X.); chenhao_sci@hunau.edu.cn (H.C.); xiaogang@hunau.edu.cn (G.X.); 2 Junlebao Dairy Co., Ltd., Shijiazhuang 050221, China; 3 Key Laboratory of Stem-Fiber Biomass and Engineering Microbiology, Institute of Bast Fiber Crops, Chinese Academy of Agricultural Sciences, Ministry of Agriculture, Changsha 410205, China

**Keywords:** rapeseed, acetylation sequencing, Western blotting, fluorescence quantification, site-directed mutation

## Abstract

**Simple Summary:**

The quality of rapeseed (*Brassica napus*) oil depends mainly on the degree of hydrocarbon chain saturation of fatty acids. In previous studies, seeds of the *B. napus* near-isogenic lines were used as raw materials by iTRAQ analysis at 20–35 days after pollination, and three differential proteins related to oleic acid metabolisms were found to be related to acetylation modification. Thus, we analyzed lysine acetylation using the same raw materials. The function of the corresponding genes of fatty acid metabolisms-related differential proteins was verified by identifying the overexpression of *BnACP3^63K^*, *BnACP3^63R^*, and *BnACP3^63^* in *Arabidopsis thaliana*. The results show that the acetylation modification of *BnaACP3* may have a selective effect on oleic acid and slow down the conversion of oleic acid to linoleic acid. This is the first report of oleic acid synthesis regulation by acetylation.

**Abstract:**

In this study, lysine acetylation analysis was conducted using two *Brassica napus* near-isogenic lines, HOCR and LOCR, containing high and low oleic acid contents, respectively, to explore this relationship. Proteins showing differences in quantitative information between the *B. napus* lines were identified in lysine acetylation analysis, and KEGG pathways were analyzed, yielding 45 enriched proteins, most of which are involved in carbon fixation in photosynthetic organisms, photosynthesis, ascorbate and aldarate metabolism, and glycolysis. Potential key genes related to fatty acid metabolisms were determined. To further explore the effect of acetylation modification on fatty acid metabolisms, the acyl-ACP3 related gene *BnaACP3^63K^* was cloned, and a base mutation at No.63 was changed via overlapping primer PCR method. This study is the first to demonstrate that acetylation modification can regulate oleic acid metabolisms, which provides a promising approach for the study of the molecular mechanism of oleic acid in rapeseed.

## 1. Introduction

Rapeseed (*Brassica napus*) is the third most important oilseed crop in the world [1], and the quality of rapeseed oil depends mainly on the degree of hydrocarbon chain saturation of fatty acids. Fatty acids are divided into three categories according to the saturation of the hydrocarbon chain: saturated fatty acids (mainly palmitic acid and stearic acid), monounsaturated fatty acids (oleic acid, erucic acid, etc.), and polyunsaturated fatty acids (PUFA) (linoleic acid, linolenic acid, etc.) [2,3]. The studies have shown that guidelines for preventing coronary heart disease focus on converting saturated and trans fats into unsaturated fatty acids in the diet [4]. The genes encoding fatty acid desaturase in plants, such as *FAD2*, *FAD3*, *FAD4*, *FAD5*, *FAD6*, *FAD7*, and *FAD8*, are also known as acyl-lipid desaturases [5]. Previous research has shown that the *FAD2* gene is crucial for oleic acid synthesis, and is associated with other genes that influence and participate in oleic acid metabolisms [6,7]. Many studies have shown that the synthesis of oleic acid may be affected by multiple minor genes [8,9]. However, current research mostly focuses on *FAD2* [10,11], and there is still a lack of research on related minor genes.

Histone Lysine acetylation (*Kac*) is a dynamic and reversible process that is important for regulating protein expression [12,13]. Under physiological conditions, lysine residues can be protonated and become positively charged, whereas acetylation can prevent the protonation of lysine residues. Therefore, removing lysine residues on the side chain of the positive charge can lead to the acetylated lysine residues exhibiting neutral amino acid properties [14]. Lysine sites are generally mutated to positively charged arginine (Arg, R) to simulate an unacetylated state and uncharged glutamine (Gln, Q) to simulate an acetylated state. When the lysine site is mutated into glutamine, the protein function changes significantly, which indicates that acetylation modification of this site plays an important role in regulating protein function [15,16,17]. In *Arabidopsis*, histone acetyltransferase has been found to regulate fatty acid biosynthesis [18,19]. The main DNA sequence of the protein-coding region is highly conserved between rapeseed and *A. thaliana* [20].

The results show that *B. napus* and *A. thaliana* are both cruciferous plants with highly similar gene functions. Thus, it is expected that acetylation modification may also affect fatty acid synthesis in *B. napus*. In previous studies, seeds of the *B. napus* near-isogenic lines were used as raw materials by iTRAQ analysis at 20–35 days after pollination, and three differential proteins related to oleic acid metabolisms were found to be related to acetylation modification [21]. This study analyzed lysine acetylation using the same raw materials. The function of the corresponding genes of fatty acid metabolism-related differential proteins was verified by identifying the overexpression of *BnACP3^63K^*, *BnACP3^63R^*, and *BnACP3^63Q^* in *A. thaliana*.

## 2. Materials and Methods

### 2.1. Materials

For this study, two *B. napus* near-isogenic lines, HOCR and LOCR, containing different oleic acid contents (81.4% and 56.2%, respectively) were provided by the College of Agronomy, Hunan Agricultural University (Changsha, China), and the lower oleic acid content (56.2%) *B. napus* was used as control. Self-pollinating (mixed the 20 d, 25 d, 30 d, and 35 d seeds after pollination) were sampled from 10 individual plants for each line, and were mixed into one biological replicate for further analysis. In total, three biological replicates were collected for each stage, immediately frozen in liquid nitrogen, and stored at −80 °C for LC-MS/MS analysis. Planting and sample collection methods were carried out as described in a previous study [22].

### 2.2. Methods

#### 2.2.1. Acetylation Modification

##### Protein Extraction

The protein extraction and peptide digestion procedures were modified from previously reported procedures [23]. Briefly, the mixed seeds (100 mg) were preserved in liquid nitrogen and ground into cell powder. The powder was then transferred to 5 mL centrifuge tubes. In each tube, four volumes of lysis buffer (8 M urea (Sigma-Aldrich, Saint Louis, MO, USA), 1% TritonX-100 (Sangon Biotech, Shanghai, China), 10 mM dithiothreitol (Sigma-Aldrich, Saint Louis, MO, USA), and 1% protease inhibitor cocktail (Merck Millipore, Billerica, MA, USA)) were added, and the tubes were sonicated three times on ice using a high intensity ultrasonic processor (Ningbo Scientz Biotechnology Co., Ltd., Ningbo, Zhejiang, China). For the post-translational modification experiments, inhibitors were added to the lysis buffer (e.g., 3 μM trichostatin A (TSA) (Med Chem Express, South Brunswick, MA, USA) and 50 mM nicotinamide (Sigma-Aldrich, Saint Louis, MO, USA) for acetylation). The remaining debris was removed by centrifugation at 20,000× *g* at 4 °C for 10 min. The protein was precipitated with cold 20% trichloroacetic acid (Sigma-Aldrich, Saint Louis, MO, USA) for 2 h at −20 °C, and the supernatant was discarded. The remaining precipitate was washed three times with cold acetone. The protein was redissolved in 8 M urea, and the protein concentration was determined using a BCA kit (Beyotime Biotechnology, Shanghai, China), according to the manufacturer’s instructions.

##### Trypsin Digestion

For protein digestion, the protein solution was reduced with 5 mM dithiothreitol for 30 min at 56 °C, and then alkylated with 11 mM iodoacetamide (Sigma-Aldrich, Saint Louis, MO, USA) for 15 min at room temperature in the dark. The protein sample was diluted by adding 100 mM triethylammonium bicarbonate (TEAB) (Sigma-Aldrich, Saint Louis, MO, USA) to a urea concentration < 2 M. Finally, trypsin (Promega, Madison, WI, USA) was added at a 1:50 trypsin-to-protein mass ratio for the first digestion for 12 h, and 1:100 trypsin-to-protein mass ratio for a second digestion of 4 h.

##### Tandem Mass Tag (TMT) Labeling

After trypsin digestion, the peptides were desalted on a Strata X C18 SPE column (Phenomenex) and vacuum-dried, reconstituted in 0.5 M TEAB, and processed according to the manufacturer’s protocol for the TMT kit (Thermo Fisher Scientific, Waltham, MA, USA). Briefly, one unit of TMT reagent was thawed and reconstituted in acetonitrile (Fisher Chemical, Waltham, MA, USA). The peptide mixtures were then incubated for 2 h at room temperature, pooled, desalted, and dried by vacuum centrifugation.

##### Liquid Chromatography Coupled with Tandem Mass Spectrometry (LC-MS/MS) Analysis

The tryptic peptides were dissolved in 0.1% formic acid (solvent A) (Sigma-Aldrich, Saint Louis, MO, USA), and directly loaded onto a homemade reversed-phase analytical column (15 cm length, 75 μm i.d.). The gradient of solvent B (0.1% formic acid in 98% acetonitrile) was increased from 6% to 23% over 26 min, 23% to 35% over 8 min, to 80% over 3 min, and then held at 80% for the last 3 min. The gradient was increased at a constant flow rate of 400 nL/min on an EASY-nLC 1000 ultra-performance liquid chromatography (UPLC) system (ThermoFisher Scientific, Waltham, MA, USA).

The peptides were subjected to a nanospray ion source, followed by tandem mass spectrometry (MS/MS) in a Q ExactiveTM Plus mass spectrometer (Thermo Fisher Scientific, Waltham, MA, USA) coupled online with the UPLC system. The applied electrospray voltage was 2.0 kV. The *m*/*z* scan range was 350–1800 for full scan, and intact peptides were detected 31/35 in the Orbitrap (Thermo Fisher Scientific, Waltham, MA, USA) at a resolution of 70,000. Peptides were selected for MS/MS using a normalized collision energy setting of 28, and the fragments were detected in the Orbitrap at a resolution of 17,500. The data-dependent procedure alternated between one MS scan followed by 20 MS/MS scans with 15 s dynamic exclusion. The automatic gain control was set at 5 × 10^4^, and the fixed first mass was set at 100 *m*/*z*.

##### Database Search

The resulting MS/MS data were processed using the MaxQuant search engine (v.1.5.2.8). Tandem mass spectra were searched against *B. napus* databases concatenated with a reverse decoy database. Trypsin/P was specified as the cleavage enzyme, allowing up to four missing cleavages. The mass tolerance for precursor ions was set as 20 ppm in the first search and 5 ppm in the main search, and the mass tolerance for fragment ions was set as 0.02 Da. Carbamidomethyl on Cys was specified as the fixed modification, and acetylation modification and oxidation on Met were specified as variable modifications. The false discovery rate was adjusted to <1%, and the minimum score for modified peptides was set as >40.

The LOCR was used as a control, and the 1.3 times change threshold was set as the standard. A *t*-test *p*-value of <0.05 was considered to be significant. Functional annotations of the differentially expressed proteins were conducted using gene ontology (GO) annotation (http://www.geneontology.org/, accessed on 4/11/2019), and proteins were categorized according to their biological processes, molecular functions, and cellular localization. The Kyoto Encyclopedia of Genes and Genomes (KEGG) database was used to classify and group the identified proteins.

#### 2.2.2. Analysis and Verification of Sequencing Results

##### Western Blot Analysis

Total proteins of HOCR and LOCR seedlings were prepared with extraction solution (50 mM Tris-HCl, 150 mM NaCl, 2% SDS, 0.05% β-mercaptoethanol, and 1 mM protease inhibitor). Proteins were separated by 12% SDS–PAGE and transferred onto a nitrocellulose filter membrane (Pall, New York, NY, USA). Antibodies against Anti-H3K27ac (PTM Biolabs) were used as the primary antibody (1:1000 dilution), and histone H3 was used as an internal control. Signals were detected using SuperSignal West Pico Plus chemiluminescent substrate (Thermo Scientific, Waltham, MA, USA).

##### Gene Expression Validation by Real-Time PCR (qPCR) Analysis

The total RNA of the two *B. napus* was extracted from the mixed seeds (100 mg) respectively, and the cDNA was synthesized according to the instructions of the Reverse Transcription Kit (TRANS, Beijing, China). The total RNA of three genes related to fatty acid metabolisms and four genes related to the tricarboxylic acid cycle were extracted from rapeseed seeds 20–35 days after pollination using a Plant RNA Midi Kit (Omega, Norcross, GA, USA). *BnaActin* (FJ529167.1) was used as the reference gene with the following primer sequences: (F) 5-GGTTGGGATGGACCAGAAGG-3 and (R) 5-TCAGGAGCAATACGGAGC-3′.

The database of *B. napus* on the Genome Browser (https://www.genoscope.cns.fr/brassicanapus/, accessed on 6/5/2020) and Brassica Database (https://brassicadb.org/brad/, accessed on 14/6/2020) were used to retrieve The qPCR analysis method was modified from previously reported procedures [24]. The relative expression level of each gene was evaluated using the comparative cycle threshold (Ct) method [25]. Data were analyzed statistically by analysis of variance and post-hoc tests, with a *p*-value < 0.05 considered significant. All primers used for qPCR analysis are listed in Table 1.

#### 2.2.3. Gene Function Verification

##### Cloning of Fatty Acid Metabolism-Related Genes and Introduction of Base-Directed Mutations

Total RNA was extracted from the leaves of HOCR *B. napus* seedlings (100 mg). cDNA was synthesized according to the instructions of the Reverse Transcription Kit. According to the results of the differential expression data of acetylation modification, the acyl carrier protein (GSBRNA2T00100854001) was cloned by PCR. In the *B. napus* database (http://www.genoscope.cns.fr/brassicanapus/)(30/6/2020), its cDNA sequence and CDS sequence were downloaded, and named *BnaACP3* (*BnaC09g16320D*) (Appendix A).

According to the sequencing results, a mutant base was introduced by the overlapping primer method at the position of amino acid 63 (lysine). We respectively converted a positively charged lysine (lysine, K, AAA) to a positively charged arginine (Arg, R, AGA) to indicate that no acetylation modification had occurred, and converted a positively charged lysine (lysine, K, AAA) to an uncharged glutamine (Gln, Q, CAA) to indicate that the acetylation modification had occurred. Using the cDNA of the HOCR seedling leaves of *B. napus* as the template, primers were designed according to the sequence and required mutation bases (Table 2).

Primers F1 and R2 were used to clone *BnaACP3^63K^* (approximately 560 bp); primers F1 and R(R)1 were used to clone *BnaACP3^63R-1^* (approximately 280 bp); and primers F(R)2 and R2 were used to clone *BnaACP3^63R-2^* (approximately 280 bp). After gel recovery and purification, these two fragments were used as templates. Then, primers F1 and R2 were used to clone *BnaACP3^63R^* (approximately 560 bp); primers F1 and R(Q)1 were used to clone *BnaACP3^63Q-1^* (approximately 280 bp); and primers F(Q)2, and R2 were used to clone *BnaACP3^63Q-2^* (approximately 280 bp). After the gel was recovered, these two products were used as templates, and primers F1 and R2 were used to clone *BnaACP3^63Q^* (approximately 560 bp).

According to the instructions of the Zero Background pTOPO-TA Simple Cloning Kit (TransGen Biotech, Beijing, China), the target fragment was linked to the T vector.

##### Overexpression Vector Construction

Primer M13 was designed to detect positive clone vectors. According to the *BnaACP3* gene sequence information, *EcoR* I (GAATTC) and *Sal* I (GTCGAC), which are near the multiple cloning site of the plant binary expression vector pCAMBIA1300, were selected as restriction sites. A sequence in the 35s promoter was selected as the upstream primer, and a conserved sequence in BnaACP3 was selected as the downstream primer, which was used as the detection primer for positive expression vectors and transgene-positive shoots. Detection primer 1 was designed based on the Hyg resistance tag sequence on the expression vector pCAMBIA1300 Detection primer 2, and all primers were designed using Primer Premier 6.0, and synthesized by Tsingke Biotechnology Co., Ltd. (Beijing, China) (Table 3).

The positive clone vector plasmids were selected and double-digested, and the plant expression vector pCAMBIA1300 was restricted by *EcoR* I and *Sal* I enzymes, respectively. After the target fragment was recovered, T4 DNA ligase was used to link the recovered target fragment with the linearized *pCAMBIA1300*. The three recombinant plasmids were transformed into DH5α using the heat shock method. The target fragment was approximately 1153 bp, and the restriction enzymes *EcoR* I and *Sal* I were used for double digestion detection.

Planting and infestation of wild-type (WT) *A. thaliana* was carried out by following a modified version of the experimental method reported by Clough et al. [26]. The harvested *A. thaliana* transgenic T0 generation seeds were screened using Hyg (50 mg/mL) + 1/2 MS solid medium, and sterilized; the T0 generation seeds were sterilized and evenly planted in Hyg (50 mg/mL) + 1/2MS solid medium and transferred to a plant growth incubator after vernalization. After 7–10 days, green seedlings were observed in the petri dish. The green seedlings with good root growth were transplanted into the nutrient soil substrate, and the seedling bowl was transferred to the plant normal culture in a growth incubator (24 °C, 16 h light).

##### Functional Verification of *Arabidopsis*

T1 generation transgenic seeds and WT seeds were collected in an EP tube and placed in an oven at 60 °C for approximately 1 week. The fatty acid composition of the seeds was determined by gas chromatography [10], and 3 repetitions were used to average.

## 3. Results

### 3.1. Acetylation Modification Results

#### 3.1.1. Quality Control Testing by Mass Spectrometry

**Figure 1:**
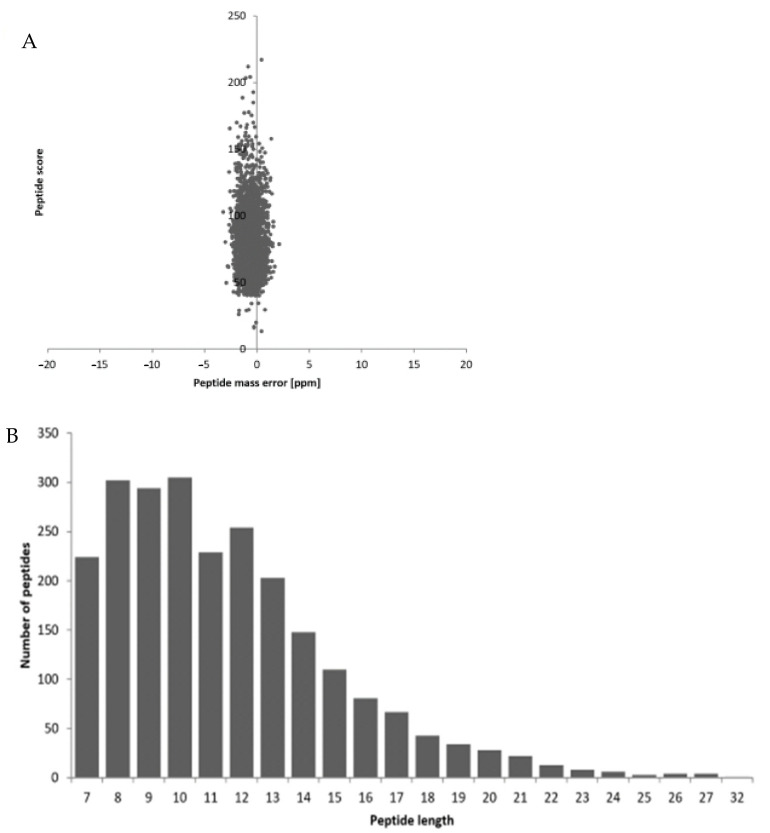
Quality control test results of mass spectral data. (**A**) Mass error and (**B**) length distribution of the identified peptides.

The sample was analyzed as described in Section 2.2.1, and the results are shown in Figure 1, where the mass error was centered at 0 and concentrated in the range below 10 ppm, indicating that the quality error meets the requirements; second, most peptide lengths were distributed between 8–20 amino acid residues, which conforms to the rule of trypsin digestion, indicating that the sample preparation meets the standard.

#### 3.1.2. Proteome-Wide Analysis of Kac Sites in Rapeseed

A total of 2903 acetylation sites were identified on 1610 proteins of which 2473 sites on 1409 proteins contained quantitative information. The modification expression of the LOCR was used as a control, and the modification sites with a quantitative ratio > 1.3 or <0.769 and *t*-test *p*-value < 0.05 were considered significantly differentially abundant. Moreover, the modification level of 80 sites was upregulated, and the modification level of 21 sites was downregulated.

#### 3.1.3. GO Analysis of Acetylated Proteins

**Figure 2:**
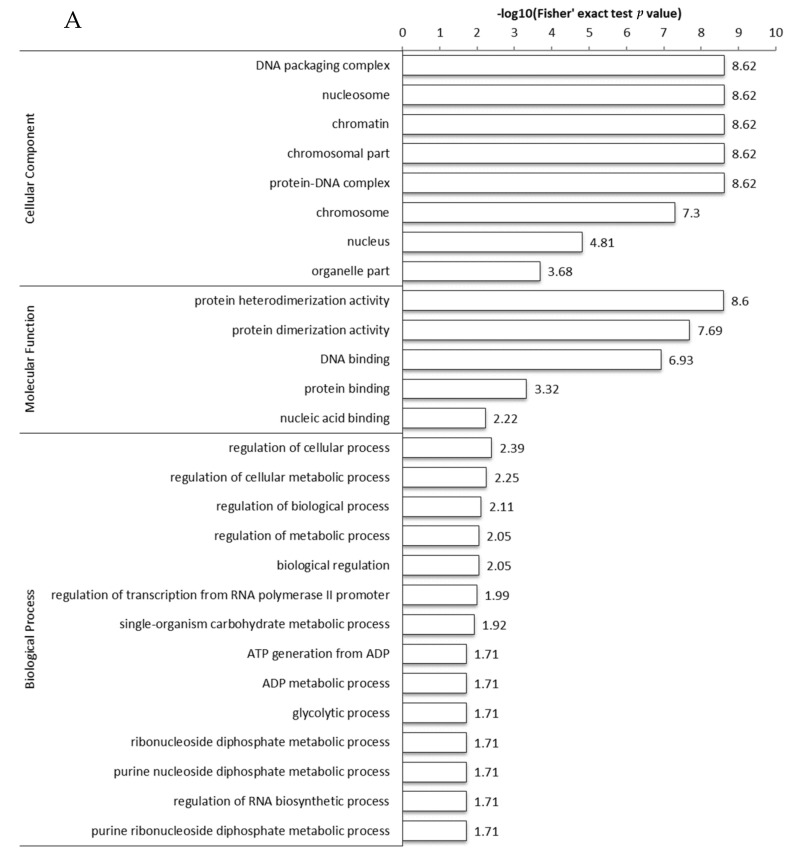

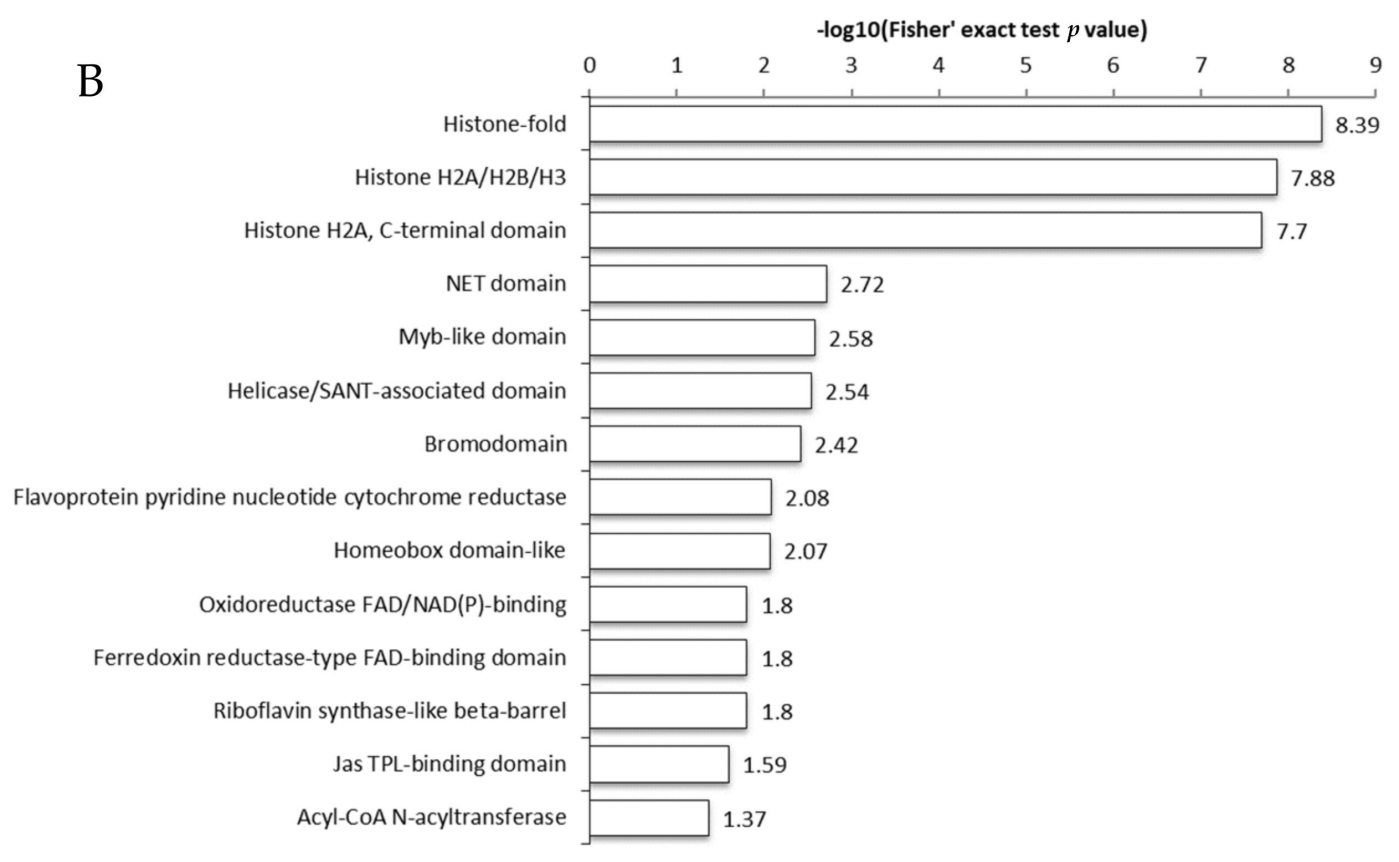
Gene ontology and domain enrichment analysis of acetylated proteins. The abscissa value is a negative logarithmic transformation of a significant *p*-value (*p* < 0.05). (**A**) GO enrichment analysis and (**B**) domain enrichment analysis of differentially expressed proteins.

To better understand the functions of acetylated modified proteins, functional enrichment analysis of molecular functions, cell composition, and biological processes were carried out (Figure 2A), and the results were converted to negative logarithms (−log10). For cell composition, DNA packaging complexes, nucleosomes, chromatin, chromosomal parts, protein-DNA complexes, chromosomes, nuclei, and organelles were significantly enriched. For molecular functions, protein heterodimerization, protein dimerization, DNA binding protein, protein binding protein, and nucleic acid binding protein were enriched; of these, the highest degree was protein heterodimerization, and the lowest was nucleic acid binding protein. For biological processes, cell process regulation, cell metabolism regulation, and biological process regulation, regulatory metabolic process, biological regulation, RNA polymerase II promoter transcription regulation, single biological carbohydrate metabolism process, ADP metabolic process, glycolysis process, ribonucleoside diphosphate metabolic process, purine nucleoside diphosphate metabolism process, RNA biosynthesis process regulation, and purine ribonucleoside diphosphate metabolism process were enriched.

In addition, domain enrichment analysis was performed for acetylated modified proteins with significant differences in sequencing (Figure 2B). A total of 14 domains, including histone folding, oxidoreductase FAD/NAD (P) binding, ferredoxin FAD-binding domain, and acyl-coenzyme A and N-acyltransferase, were significantly enriched in the identified proteins.

#### 3.1.4. Protein Acetylation Regulates Diverse Metabolic Pathways in Rapeseed

**Figure 3:**
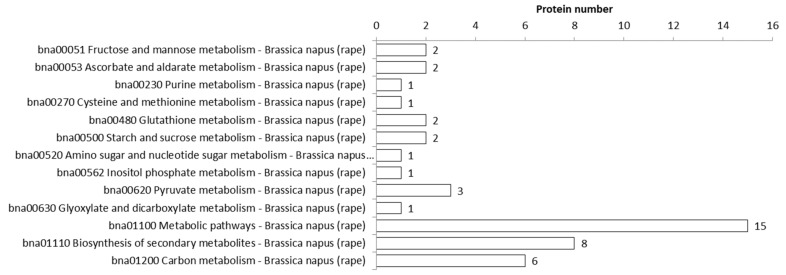
Analysis of multiple metabolic pathways.

In this study, KEGG metabolic enrichment analysis was performed on the identified differentially acetylated proteins, and it was found that proteins related to metabolic pathways such as photosynthesis, carbon metabolism, citrate pyruvate metabolism, glyoxylic acid, and dicarboxylic acid metabolism were significantly enriched (Figure 3), and 3-oxoacyl-(acyl-carrier protein) synthase II (FABF), acyl-(acyl-carrier protein) desaturase (FAB2), and acyl-ACP3 were related to fatty acid metabolisms. The results indicate that the acetylation modification of the *B. napus* protein may be involved in regulating multiple metabolic pathways.

### 3.2. Verification Analysis of Sequencing Result

#### 3.2.1. Validation of Protein Expression by Western Blot

Histone H3K27ac (GSBRNA2T00135897001) was used to quantitatively verify the Western blot results for high and low oleic acid rapeseed. The results demonstrate that the abundance of the same protein was more acetylated in HOCR, which is consistent with the results of the acetylation analysis (Figure S1).

#### 3.2.2. Gene Expression Validation by qPCR

The expressions of three genes that encode proteins involved in fatty acid metabolism (acyl-(acyl-carrier-protein) desaturase 5,3-oxoacyl-(acyl-carrier-protein) synthase I, acyl carrier protein 3), and four genes related to the tricarboxylic acid cycle (phosphoglycerate kinase 1, probable fructose-bisphosphate aldolase, triosephosphate isomerase, and plastidial pyruvate kinase) were verified (Table 1).

**Figure 4:**
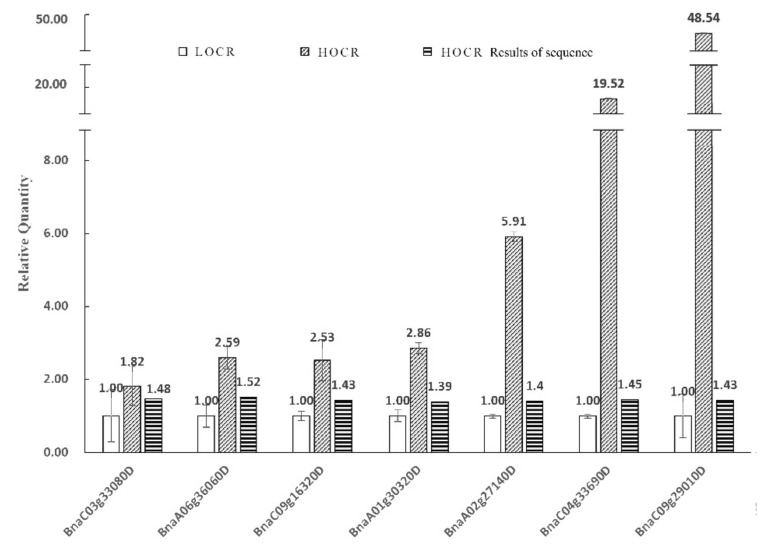
Gene expression of seeds 20–35 d after self-pollination. Note: Legend HOCR results means the modification expression of the LOCR was used as a control, and the modification sites with a quantitative ratio > 1.3 and *t*-test *p*-value < 0.05 were considered significantly differentially.

Compared with low oleic acid rapeseed, high oleic acid rapeseed showed upregulated transcript levels of acyl-(acyl-carrier-protein) desaturase 5 (1.82-fold), 3-oxoacyl-(acyl-carrier-protein) synthase I (2.59 fold), acyl carrier protein 3 (2.53 fold), phosphoglycerate kinase 1 (2.86 fold), probable fructose-bisphosphate aldolase 3 (5.91 fold), triosephosphate isomerase (19.52 fold), and plastidial pyruvate kinase (48.54 fold) (Figure 4). These results are consistent with the sequencing results.

### 3.3. Gene Function Verification

#### 3.3.1. Cloning of Fatty Acid Metabolism-Related Genes and Introduction of Base-Directed Mutations

Using this as the template, a mutant base was introduced at position 63 to change the amino acid via the overlapping primer PCR method. *BnaACP3^63R-1^*, *BnaACP3^63R-2^*, *BnaACP3^63Q-1^*, and *BnaACP3^63Q-2^* were cloned, which were approximately 280 bp in length (Figure S2A). *BnaACP3^63R^* (approximately 560 bp) was synthesized using *BnaACP3^63R-1^* and *BnaACP3^63R-2^* as templates, and *BnaACP3^63Q^* (approximately 560 bp) were synthesized using *BnaACP3^63Q-1^* and *BnaACP3^63Q-^*^2^ as templates (Figure S2B).

**Figure 5:**
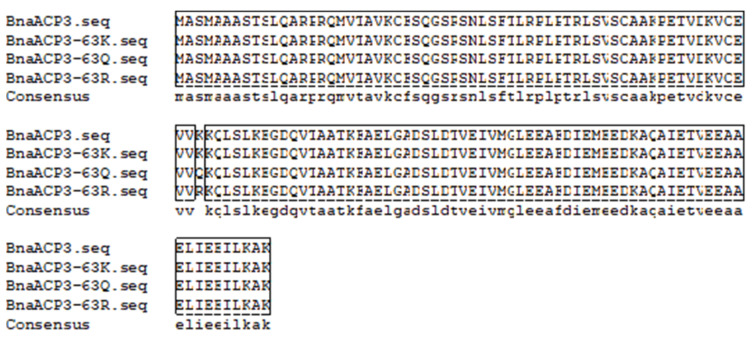
The comparison of amino acid sequences of the three genes after mutation. Note: The amino acid at position 63 was successfully mutated.

The recovered target gene fragment was linked to the pTOPO-T simple vector and transformed into Trans1-T1 competent cells. After single colony screening (approximately 700 bp) (Figure S3) and colony PCR verification, the positive recombinants were obtained and sent to Tsingke Biotechnology Co., Ltd. for sequencing (Figure 5). The results show that the amino acid at position 63 was mutated into arginine and glutamine by overlapping PCR primers, and the expected results were obtained.

#### 3.3.2. Overexpression Vector Construction and *Arabidopsis* Transformation

The results are shown in Figure S4. The size of the electrophoresis fragment was consistent with that of the target fragment after plasmid PCR detection and enzyme digestion detection, indicating that the *pCAMBIA 1300-BnaACP3^63K^*, *pCAMBIA 1300-BnaACP3^63R^*, and *pCAMBIA 1300-BnaACP3^63Q^* vector were successfully constructed.

The successfully constructed plant expression vector were transformed into competent *Agrobacterium* tumefaciens GV3101 cells using the heat shock method, respectively. PCR detection was performed using detection primer 1 (1153 bp). The *pCAMBIa130-BnaACP3* plant expression vector was successfully transformed into A. tumefaciens (Figure S5), and transferred to *A. thaliana*. A 50 mg/mL KanR-resistant plate was used to screen T0 generation seeds, and the RNA was extracted from transgenic *Arabidopsis* leaves, and cDNA was synthesized by reverse transcription. Primer 1 was used for molecular identification (Figure S6).

#### 3.3.3. Fatty Acid Composition Analysis in Transgenic *Arabidopsis*

**Figure 6:**
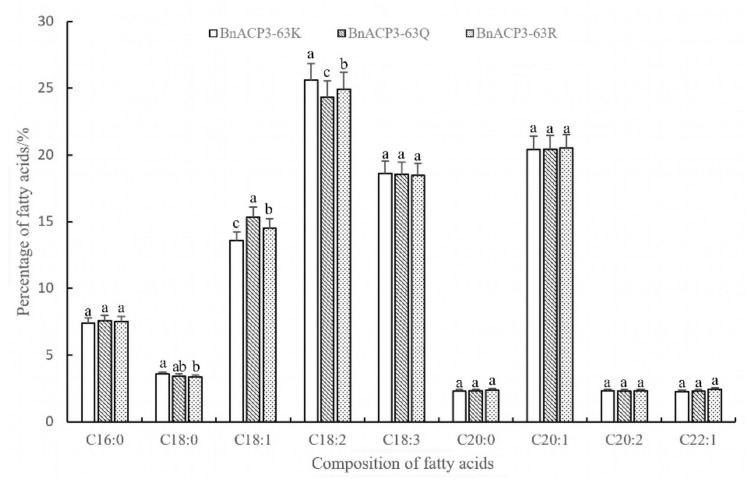
Fatty acid composition analysis of transgenic *Arabidopsis* seeds. Note: The Spss 22.0 was used for statistical analysis, and different letters indicated significant difference at the same period (*p* < 0.05). According to the principle of statistics, different letters mean significant differences, while the same letters mean insignificant differences.

The fatty acid composition of the transgenic T1 generation seeds of *BnaACP3^63K^*(CK) *BnaACP3^63Q^*, and *BnaACP3^63R^* were analyzed by gas chromatography, and the results are shown in Figure 6. Compared with CK, *BnaACP3^63Q^* and *BnaACP3^63R^* genes of *B. napus* were heterologously expressed in *A. thaliana*. The oleic acid (C18:1) content of simulated acetylated *BnaACP3^63Q^* was approximately 1.75% higher than that of CK, with a range of 12.9%. Linoleic acid (C18:2) content decreased by 1.27% and 4.95%, compared with CK; the content of oleic acid in the simulated *BnaACP3^63R^* that did not undergo acetylation modification increased by approximately 0.91%, with an amplitude of 6.67%, while the content of linoleic acid decreased by 0.68%, with an amplitude of 2.64%. This indicates that the acetylation of *BnaACP3* may have a selective effect on oleic acid and linoleic acid, and can slow down the conversion of oleic acid to linoleic acid.

## 4. Discussion

Proteins are effectors of biological function, and their levels are not only dependent on corresponding mRNA levels, but also on host translational control and regulation. Thus, the proteomics would be considered as the most relevant data set to characterize a biological system [27]. In this study, a group of near-isogenic lines of *B. napus* with different oleic acid contents were used as materials for acetyl sequencing, and the accuracy and reliability of the sequencing results were verified by the protein and gene expression levels.

The palmitic acid (C16:0) and stearic acid (C18:0) are the most commonly consumed saturated fatty acids in the diet of people [28]. It is generally believed that palmitic acid can improve cholesterol more than stearic acid [29,30,31]. When the mass fraction of linolenic acid is not less than 5%, the lower the saturated fatty acid content, the higher the oleic acid content. The natural edible vegetable oils with higher omega-6 and omega-3 ratios of 1~4:1 have the most nutritional value [2]. Therefore, rapeseed oil quality improvement has become a hot spot in the field of *B. napus* breeding.

Acyl carrier proteins are a complex group of small proteins that play an important role in cellular metabolism, and are located in the center of the fatty acid metabolism pathway [32]. The study of the ACP gene in plants has been progressing rapidly. Overexpression of ACP-1 in *Arabidopsis* leaf could increase the content of linolenic acid (18:3), and decreases the content of fatty acid (16:3) in leaves [33]. The expression of *ACP4* was significantly reduced by the antisense RNAi, resulting in a decrease in leaf fatty acid content and a decrease in total fatty acid composition [34]. Therefore, altering the expression of the ACP gene may cause a change in the composition and content of fatty acids in rapeseed oil. In this study, *BnaACP3^63K^* was cloned, and *BnaACP3^63R^* and *BnaACP3^63Q^* were obtained by introducing a base mutation into No. 63 by overlapping PCR primers. Analysis of the fatty acid composition of the T1 generation showed that the acetylation modification of *BnaACP3* might have a selective effect on oleic acid and linoleic acid, and can hinder the conversion of oleic acid to linoleic acid. *A. thaliana* and *B. napus* belong to the same family, *Brassicaceae*, and share the same primitive ancestors. The study of *A. thaliana* can provide an important reference for research on *B. napus* [35,36].

This paper reports the first discovery of the effect of acetylation modification on fatty acid metabolisms, and provides a reference for further study on fatty acid metabolisms in rapeseed. Furthermore, the methods used in this study may be applied to determine the lysine acetylation landscape in other plants.

## 5. Conclusions

The Kac sites in the whole proteome of seeds 20–35 days after pollination were identified in two *B. napus* near-isogenic lines, and the gene functions of key differential proteins were determined. A total of 2903 acetylation sites and 1610 proteins were identified. Among them, 2473 sites in 1409 proteins contained quantitative information. In the quantified acetylation sites, the modification levels of 80 sites in the HOCR/LOCR comparisons were upregulated, and those of 21 sites were downregulated. Moreover, it was found that FABF, FAB2, and acyl-ACP3 were related to fatty acid metabolisms. The sequencing samples sent in the same batch were used as materials, and WB and qPCR validation were used to verify the accuracy and reliability of the sequencing results. The *BnaACP3^63K^* gene was cloned from HOCR seedling leaves of *B. napus*, and a base mutation was introduced at position 63 by the overlapping primer PCR method. In total, two amino acid-directed mutations, *BnaACP3^63R^* and *BnaACP3^63Q^*, were obtained. The overexpression vectors of these three genes were constructed and transformed into *A. thaliana* to verify the effect of acetylation modification on fatty acid metabolisms. The results show that the acetylation modification of *BnaACP3* may have a selective effect on oleic acid, and slow down the conversion of oleic acid to linoleic acid. This is the first report of oleic acid synthesis regulation by acetylation. The gene function will be verified in rapeseed, and the function of acetylation will be carried out in the field.

## Figures and Tables

**Table 1:** Expression level of corresponding genes of acetylation-related differential proteins.

Differential Proteins	Codes of Corresponding Proteins	Corresponding Gene in *B. napus*	Primer Sequences (5′ to 3′)
acyl-(acyl-carrier-protein) desaturase 5	GSBRNA2T00153661001	*BnaC03g33080D*	F:TTCGTGGTGCTTGTTGGT R:GGGTTGTTCTCAGTTTTAGG
3-oxoacyl-(acyl-carrier-protein) synthase I	GSBRNA2T00054708001	*BnaA06g36060D*	F:GGACTGGTATGGGTGGTTT R:GGTAGCACAAGCGGTAGAG
acyl carrier protein 3	GSBRNA2T00100854001	*BnaC09g16320D*	F:GTTCTTCACCCTCCTCTCTTTG R:GCTTTTTGACCACTTCACACACT
phosphoglycerate kinase 1	GSBRNA2T00076479001	*BnaA01g30320D*	F:ACAATCACTGACGATACGAGG R:TGGACAGGATGACTTTAGCAC
probable fructose-bisphosphate aldolase	GSBRNA2T00069603001	*BnaA02g27140D*	F:CTTTCGTCTGGCGGAGTCTTC R:GCAATCGTTTTGGCGGTTT
triosephosphate isomerase	GSBRNA2T00108116001	*BnaC04g33690D*	F:TCATCTATCCGTCTCGTTTC R:GAGTCCTTAGTCCCGTTACA
plastidial pyruvate kinase	GSBRNA2T00009340001	*BnaC09g29010D*	F:ATGGCTCAGGTGGTTGCT R:CCTCTTCGCTTCGTTTCC

**Table 2:** Primers used for cloning BnACP3 genes and site mutation.

Primer Name	Primer Sequence (5′→3′)
F1	GAATTCTCCTCTCTTTGCCTTTCTCCGC
R(R)1	AGTTGCTTTCTGACCACTTCACACA
F(R)2	TGTGTGAAGTGGTCAGAAAGCAACT
R2	GTCGACGTGGGTTTGGGTTTAGTGGGGTT
R(Q)1	AGTTGCTTTTGGACCACTTCACACA
F(Q)2	TGTGTGAAGTGGTCCAAAAGCAACT

Note: The underline indicates the restriction site, and shading indicates the site of site-directed mutation.

**Table 3:** Primers used for expression vector construction and analysis.

Primer Name	Sequence (5′→3′)	PCR Length (bp)
*BnaACP3*-Fw	GAATTCTCCTCTCTTTGCCTTTCTCCGC	536
*BnaACP3*-Rw	GTCGACGTGGGTTTGGGTTTAGTGGGGTT	
M13-Fw	TGTAAAACGACGGCCAGT	667
M13-Rv	CAGGAAACAGCTATGACC	
Detection1-Fw (35s)	AGTGGGATTGTGCGTCAT	1153
Detection1-Rv	TCAGGCGGGTAGGAAGA	
Detection2-Fw (Hyg)	GCTCCATACAAGCCAACC	670
Detection2-Rv	AGCGTCTCCGACCTGAT	

Note: The underline indicates the restriction site, hygromycin (Hyg).

## Data Availability

The data are not publicly available yet as some data sets are being used for additional publications.

## Supplementary Materials

The following supporting information can be downloaded at: https://www.mdpi.com/article/10.3390/biology11040483/s1, Figure S1: Western blot with H3K27ac antibody. 1: 20 μg protein/lane; 2: SDS-PAGE concentration: 15%; 3: Antibody H3: Abbkine, Primary antibody: Anti-H3K27ac An-tibody 1:1000 dilution; 4: 2nd antibody, Thermo, Pierce, Goat anti-Rabbit IgG, (H+L), peroxidase conjugated, 1:5000 di-lution; 5: Thermo, Pierce, goat anti-mouse IgG, (H+L), peroxidase conjugated, 1:5000 dilution; Figure S2: PCR electropherogram. (A) Segmented gene cloning electrophoresis; (B) gene cloning of *BnaACP3^63K^*, *BnaACP3^63R^*, and *BnaACP3^63Q^*; Figure S3: PCR single colony detection; Figure S4: Recombinant plasmid detection. (A) Recombinant plasmid PCR detection, lane 1–3: K, lane 4–5: R; lane 6–7, Q; (B) double enzyme digestion detection of recombinant plasmid, lane 1–2: K, lane 3–4: R, lane 5–6: Q, Lane 7: not cut; Figure S5: PCR test of colonies. Lane 1–3: *BnaACP3^63K^*; lane 4–6: *BnaACP3^63R^*; lane 7–9: *BnaACP3^63Q^*; Figure S6: PCR test of resistant plants. Lane 1–6: *BnaACP3^63K^*; lane 7–13: *BnaACP3^63R^*; lane 14–21: *BnaACP3^63Q^*.

## Appendix A

cDNA sequence and CDS sequence of BnACP3

cDNA sequence:

>BnaC09g16320D

ATATTTAAAATATATATTTTTTGATTCAATTTGAATTTTTTTCATATGAGTTTGTTTATTTTCTTTTTTCTTCTCTAATTGACCTAAATTGTATATTTTTCCTACCTGGATCTGCCAAGTACCTACTCCACCTGATTTGTTTGCATTCTCGTTGGAATCTCAGAAA

GACAGTGCTATAAAAAAAACTCTTTTATCACTTGTTCTTCACCCTCCTCTCTTTGCCTTTCTCCGCCACCCCCGTCTCACTCCTACCTCTACCTCTCTCTCTCTCTCTCTCTCTCTCCTTAGGATTCATGGCTTCCATGGCTGCTGCCTCTACTTCCCTGCAGGCTC

GTCCTCGCCAAATGGTAACTGCGGTTAAATGTTTTAGCCAGGGAAGCAGAAGCAATCTTTCTTTTACGCTTCGCCCTCTTCCTACCCGCCTGAGCGTCTCTTGCGCGGCAAAACCTGAGACAGTGGACAAAGTGTGTGAAGTGGTCAAAAAGCAACTCTC

ACTTAAAGAGGGCGACCAAGTTACGGCTGCCACCAAATTTGCTGAACTTGGTGC

TGATTCTCTTGATACGGTGGAGATTGTGATGGGCCTGGAGGAAGCGTTCGATATAGAAATGGAGGAGGACAAAGCACAGGCGATTGAGACAGTCGAGGAAGCAGCTGAGCTCATTGAGGAGATTTTGAAAGCAAAGGCTTAAGTCCAAATCATTTATTAC

AAAACCAAACGATGGAAACCCCACTAAACCCAAACCCACTGTCCTGTTATTGTT

TGGTTAGCTAGATACCACTTAATGTTTGTTGAGATTTATGTTCGTTTGGGCAAAA

ATATTACAAGGCTTGTATTTGACTTTTTCATTTTAAAACTTTTTTAGCTCCATTCTC

CACAAGTCACCTTAC

CDS sequence:

>BnaC09g16320D

ATGGCTTCCATGGCTGCTGCCTCTACTTCCCTGCAGGCTCGTCCTCGCCAAATGGTAACTGCGGTTAAATGTTTTAGCCAGGGAAGCAGAAGCAATCTTTCTTTTACGCTTCGCCCTCTTCCTACCCGCCTGAGCGTCTCTTGCGCGGCAAAACCTGAGACAGTGGACAAAGTGTGTGAAGTGGTCAAAAAGCAACTCTCACTTAAAGAGGGCGA

CCAAGTTACGGCTGCCACCAAATTTGCTGAACTTGGTGCTGATTCTCTTGATACGGTGGAGATTGTGATGGGCCTGGAGGAAGCGTTCGATATAGAAATGGAGGA

GGACAAAGCACAGGCGATTGAGACAGTCGAGGAAGCAGCTGAGCTCATTGAGGAGATTTTGAAAGCAAAGGCTTAA
