# Supplementary material for: Promising Novel Method of Acetylation Modification for Regulating Fatty Acid Metabolism in Brassica napus L
Source: Biology (Basel). 2022 Mar 22;11(4):483. doi: 10.3390/biology11040483 (PMC9029296; doi:10.3390/biology11040483)
Supplement: Supplementary file 1 [file biology-11-00483-s001.zip › biology-1592468-supplementary.pdf]

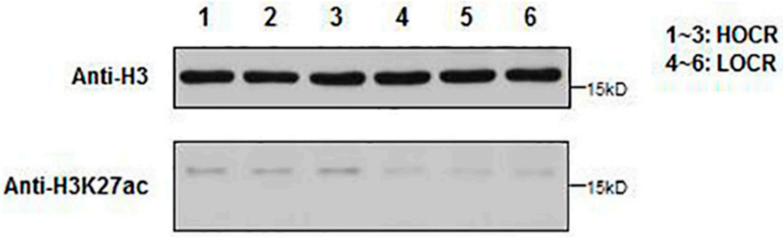

Figure S1. Western blot with H3K27ac antibody.

1: 20 μg protein/lane; 2: SDS-PAGE concentration: 15%; 3: Antibody H3: Abbkine, Primary antibody: Anti-H3K27ac Antibody 1:1000 dilution; 4: 2nd antibody, Thermo, Pierce, Goat anti-Rabbit IgG, (H+L), peroxidase conjugated, 1:5000 dilution; 5: Thermo, Pierce, goat anti-mouse IgG, (H+L), peroxidase conjugated, 1:5000 dilution

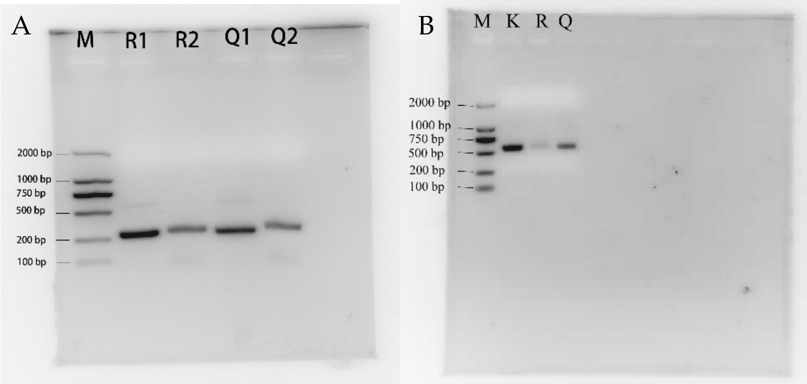

Figure S2. PCR electropherogram.

(A) Segmented gene cloning electrophoresis; (B) gene cloning of *BnaACP3*^{63K}, *BnaACP3*^{63R}, and *BnaACP3*^{63Q}.

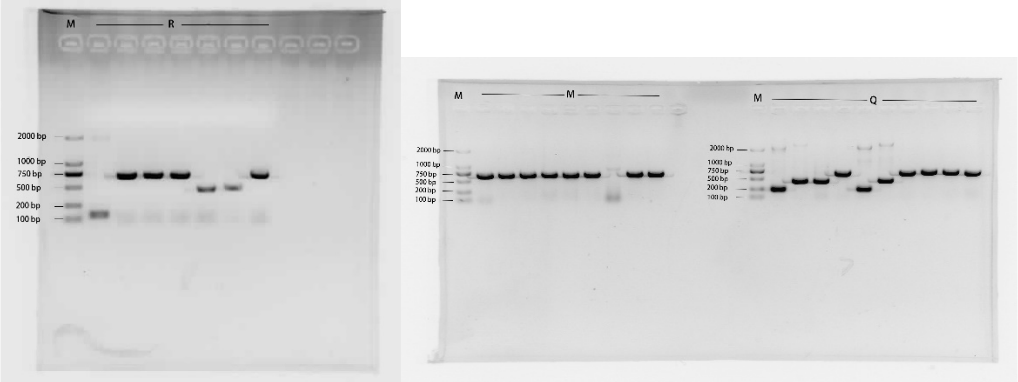

Figure S3. PCR single colony detection.

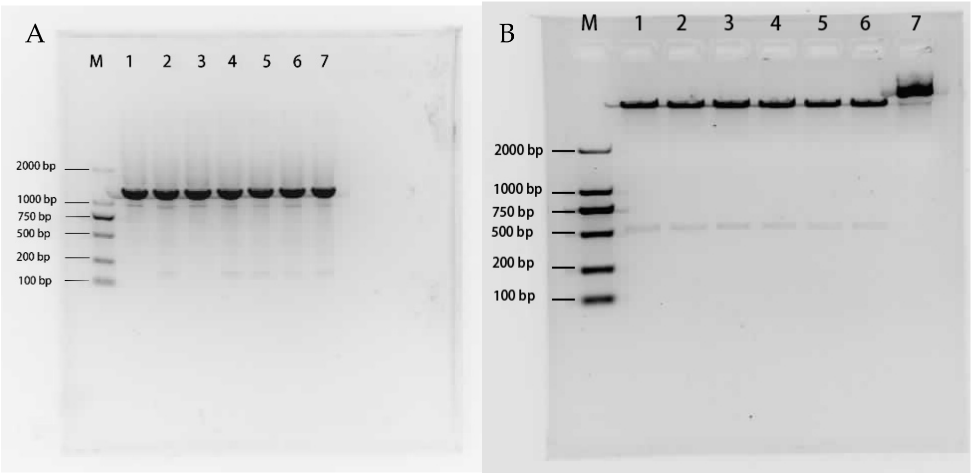

Figure S4. Recombinant plasmid detection.

Recombinant plasmid PCR detection, lane 1–3: K, lane 4–5: R; lane 6–7, Q; (B) double enzyme digestion detection of recombinant plasmid, lane 1–2: K, lane 3–4: R, lane 5–6: Q, Lane 7: not cut.

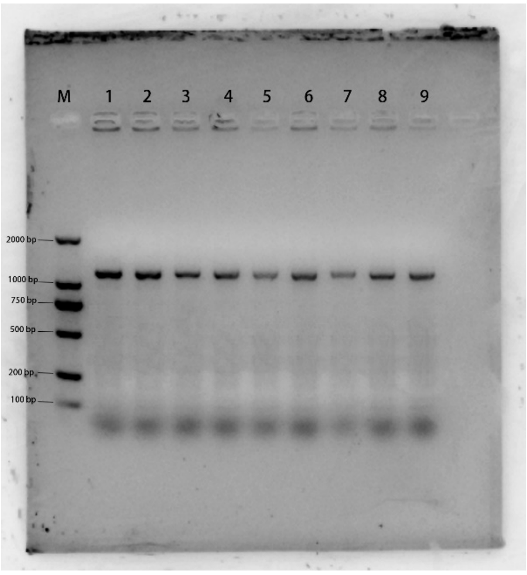

Figure S5. PCR test of colonies.

Lane 1–3: *BnaACP3*^{63K}; lane 4–6: *BnaACP3*^{63R}; lane 7–9: *BnaACP3*^{63Q}.

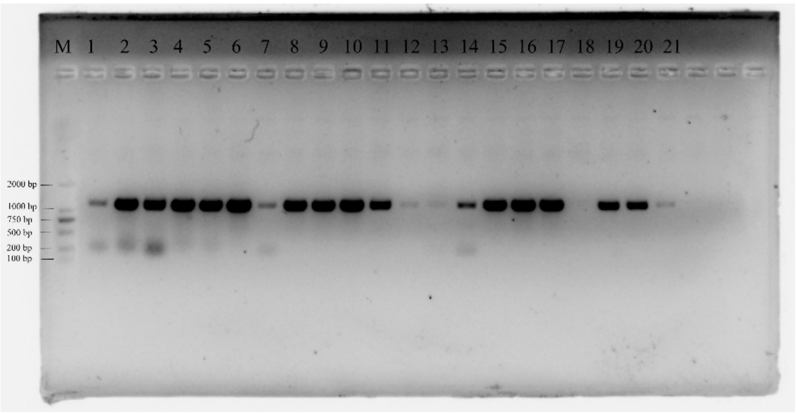

Figure S6. PCR test of resistant plants.

Lane 1–6: *BnaACP3*^{63K}; lane 7–13: *BnaACP3*^{63R}; lane 14–21: *BnaACP3*^{63Q}.
